# The solid solution Na_0.39_(NH_4_)_1.61_SO_4_·Te(OH)_6_

**DOI:** 10.1107/S1600536808003425

**Published:** 2008-02-06

**Authors:** Lilia Ktari, Mohamed Abdelhedi, Mohamed Dammak, Alain Cousson, Abdelwaheb Kolsi

**Affiliations:** a Laboratoire de Chimie Inorganique, Faculté des Sciences de Sfax, 3018 Sfax, Tunisia; b Laboratoire Léon Brillouin, CE Saclay, Bâtiment 563, 91191 Gif-sur-Yvette Cedex, France

## Abstract

The title compound, sodium ammonium sulfate–telluric acid (1/1), Na_0.39_(NH_4_)_1.61_SO_4_·Te(OH)_6_, is isostructural with other solid solutions in the series *M*_1−*x*_(NH_4_)_*x*_SO_4_·Te(OH)_6_, where ammonium is partially replaced with an alkali metal (*M* = K, Rb or Cs). The structure is composed of planes of Te(OH)_6_ octa­hedra alternating with planes of SO_4_ tetra­hedra. The Na^+^/NH_4_^+^ cations are statistically distributed over the same position and are located between the planes. The structure is stabilized by O—H⋯O and N—H⋯O hydrogen bonds between the telluric acid adducts and the O atoms of sulfate groups, and between the ammonium cations and O atoms, respectively. Both Te atoms lie on centres of symmetry.

## Related literature

For the sodium end-member of the solid solution series Na_1−*x*_(NH_4_)_*x*_SO_4_·Te(OH)_6_, see: Zilber *et al.* (1980 ▶). For the ammonium end-member of the same series, see: Zilber *et al.* (1981 ▶). For other solid solutions in the system *M*_1−*x*_(NH_4_)_*x*_SO_4_·Te(OH)_6_, where ammonium is partially replaced by an alkali metal, see: Dammak *et al.* (2005 ▶) for *M* = Cs; Ktari *et al.* (2002 ▶) for *M* = Rb; and Ktari *et al.* (2004 ▶) for *M* = K. For related literature, see: Prince (1982 ▶); Watkin (1994 ▶).

## Experimental

### 

#### Crystal data

Na_0.39_(NH_4_)_1.61_SO_4_·Te(OH)_6_
*M* *_r_* = 357.22
Monoclinic, 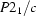

*a* = 13.690 (1) Å
*b* = 6.592 (1) Å
*c* = 11.345 (1) Å
β = 106.58 (1)°
*V* = 981.26 (19) Å^3^
*Z* = 4
Mo *K*α radiation
μ = 3.30 mm^−1^
*T* = 298 K
0.15 × 0.14 × 0.10 mm

#### Data collection

Nonius KappaCCD diffractometer
Absorption correction: multi-scan (*MULABS* in *PLATON*; Spek, 2007 ▶) *T*_min_ = 0.615, *T*_max_ = 0.719
919 measured reflections
849 independent reflections
638 reflections with *I* > 3σ(*I*)
*R*_int_ = 0.000

#### Refinement

*R*[*F* ^2^ > 2σ(*F* ^2^)] = 0.032
*wR*(*F* ^2^) = 0.043
*S* = 0.93
638 reflections
104 parameters
1 restraint
H-atom parameters constrained
Δρ_max_ = 0.51 e Å^−3^
Δρ_min_ = −1.15 e Å^−3^

### 

Data collection: *COLLECT* (Nonius, 2001 ▶); cell refinement: *DENZO*/*SCALEPACK* (Otwinowski & Minor, 1997 ▶); data reduction: *DENZO*/*SCALEPACK*; program(s) used to solve structure: *SHELXS86* (Sheldrick, 2008 ▶); program(s) used to refine structure: *CRYSTALS* (Betteridge *et al.*, 2003 ▶); molecular graphics: *DIAMOND* (Brandenburg & Berndt, 1999 ▶); software used to prepare material for publication: *CRYSTALS*.

## Supplementary Material

Crystal structure: contains datablocks I, global. DOI: 10.1107/S1600536808003425/wm2171sup1.cif

Structure factors: contains datablocks I. DOI: 10.1107/S1600536808003425/wm2171Isup2.hkl

Additional supplementary materials:  crystallographic information; 3D view; checkCIF report

## Figures and Tables

**Table 1:** Selected bond lengths (Å)

Te1—O1	1.903 (6)
Te1—O2	1.905 (4)
Te1—O3	1.916 (3)
Te2—O4	1.914 (3)
Te2—O5	1.915 (4)
Te2—O6	1.904 (5)
S1—O7	1.486 (6)
S1—O8	1.485 (3)
S1—O9	1.474 (3)
S1—O10	1.460 (6)
Na1—O6^i^	2.873 (4)
Na1—O4^ii^	2.937 (6)
Na1—O5^iii^	2.947 (4)
Na1—O3^iv^	2.950 (4)
Na1—O7^v^	2.978 (7)
Na1—O10^vi^	3.008 (4)
Na1—O9	3.120 (4)
Na1—O6^ii^	3.267 (6)
Na1—O5^vii^	3.278 (5)
Na2—O9	2.938 (5)
Na2—O8^vi^	2.966 (4)
Na2—O4^ii^	3.029 (4)
Na2—O10^viii^	3.037 (7)
Na2—O2^ix^	3.050 (4)
Na2—O2^x^	3.063 (5)
Na2—O1^xi^	3.144 (5)
Na2—O3^vii^	3.164 (5)
Na2—O1^iv^	3.305 (6)

Symmetry codes: (i) 
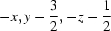
; (ii) 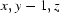; (iii) 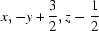; (iv) 
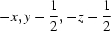
; (v) 
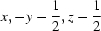
; (vi) 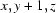; (vii) 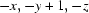; (viii) 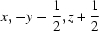; (ix) 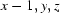; (x) 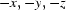; (xi) 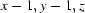.

**Table 2:** Hydrogen-bond and short contact geometry (Å, °)

*D*—H⋯*A*	*D*—H	H⋯*A*	*D*⋯*A*	*D*—H⋯*A*
O2—H2⋯O9^xii^	0.924 (4)	1.787 (4)	2.700 (6)	169.2 (2)
O3—H3⋯O8^xiii^	0.985 (5)	1.871 (5)	2.799 (7)	155.7 (2)
O4—H4⋯O7^xiv^	0.937 (3)	1.798 (3)	2.706 (7)	162.4 (2)
O6—H6⋯O10^xiii^	0.963 (4)	1.706 (4)	2.658 (6)	169.5 (3)
N1⋯.O6^i^			2.873 (4)	
N1⋯O4^ii^			2.937 (6)	
N1⋯O5^iii^			2.947 (4)	
N1⋯O3^iv^			2.950 (4)	
N1⋯O7^v^			2.978 (7)	
N1⋯O10^vi^			3.008 (4)	
N2⋯O9			2.938 (5)	
N2⋯O8^vi^			2.966 (4)	
N2⋯O4^ii^			3.029 (4)	
N2⋯O10^viii^			3.037 (7)	
N2⋯O2^ix^			3.050 (4)	
N2⋯O2^x^			3.063 (5)	

Symmetry codes: (i) 
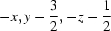
; (ii) 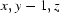; (iii) 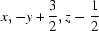; (iv) 
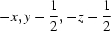
; (v) 
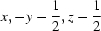
; (vi) 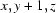; (viii) 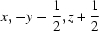; (ix) 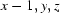; (x) 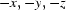; (xii) 
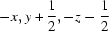
; (xiii) 
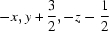
; (xiv) 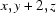.

**Fig. 1.**
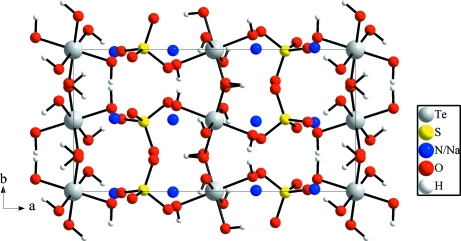
Projection of the crystal structure of the title compound on the ab plane.

**Fig. 2.**
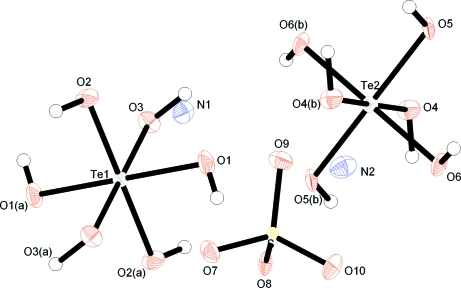
A part of the structure of Na0.39(NH4)1.61SO4.Te(OH)6, with displacement ellipsoids drawn at the 50% probability level. [Symmetry codes:(a) -x + 1,-y, -z; (b) -x, -y + 1, -z]

**Fig. 3.**
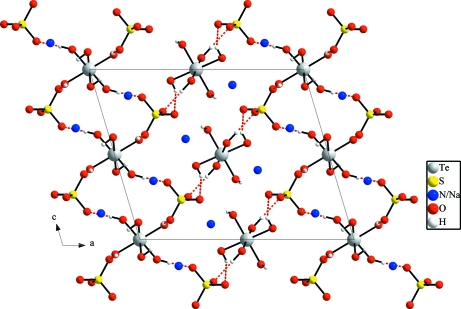
Crystal structure of Na0.39(NH4)1.61SO4.Te(OH)6 showing hydrogen bonds with dashed lines.

## supplementary crystallographic information

### Comment

The studies of a partial cationic substitution on symmetry and physical properties of solid solutions in the series *M*_1 - _*_x_*(NH_4_)*_x_*SO_4_.Te(OH)_6_ (*M* = K, Rb and Cs) have been reported in previous communications, *viz*. for K_0.84_(NH_4_)_1.16_SO_4_.Te(OH)_6_ (Ktari *et al.*., 2004), Rb_1.12_(NH_4_)_0.88_SO_4_.Te(OH)_6_ (Ktari *et al.*., 2002), and Cs_0.86_(NH_4_)_1.14_SO_4_.Te(OH)_6_ (Dammak *et al.*., 2005). To continue these studies, we have now investigated the solid solution Na_0.39_(NH_4_)_1.61_SO_4_.Te(OH)_6_. This compound is isostructural with the aforementioned phases.

Fig. 1 shows a projection of the structure on the *ab* plane. The structure can be regarded as being built up of planes of Te(OH)_6_ octahedra (at *x* = 0 and 1/2) alternating with planes of SO_4_ tetrahedra (at *x*≈ 1/4). The statistically disordered Na^+^/NH_4_^+^ cations are intercalated between these planes. Both Te atoms are situated on inversion centres and exhibit similar Te(OH)_6_ octahedra, with Te—O distances and O—Te—O angles ranging from 1.903 (6) to 1.916 (3) Å, and from 87.6 (2) to 92.4 (2)°, respectively (Fig. 2). In the sodium end-member Na_2_SO_4_.Te(OH)_6_ (Zilber *et al.*, 1980), the Te—O distances range from 1.879 (4) to 1.932 (3) Å, whereas in the ammonium end-member (NH_4_)_2_SO_4_.Te(OH)_6_ (Zilber *et al.*, 1981) they vary from 1.874 (3) to 1.944 (3) Å. The SO_4_ tetrahedra in the title compound are quite regular with S—O distances between 1.460 (6) and 1.486 (6)Å and O—S—O angles between 108.6 (3) and 110.6 (3)°. In the sodium end-member, the S—O distances are nearly the same (1.461 (5) to 1.497 (5) Å), whilst in the ammonium end-member they spread between 1.373 (11) and 1.565 (8) Å. In the mixed solution the Na/N atoms are 9-coordinate with (Na/N)—O bonds ranging from 2.873 (4) to 3.278 (5)Å for Na_1_/N_1_ and from 2.938 (5) to 3.305 (6)Å for Na_2_/N_2_. Thereby every cation is coordinated by three oxygen atoms belonging to SO_4_ tetrahedra and by six oxygen atoms belonging to Te(OH)_6_ octahedra. The structure of the title compound is stabilized *via* medium-strong O—H···O hydrogen bonds between the Te(OH)_6_ octahedra and SO_4_ tetrahedra (Fig. 3), and between N—H···O hydrogen bonds between the ammonium cations and various oxygen atoms in the structure (see hydrogen bonding Table).

### Experimental

Transparent, colorless single crystals of the title compound were grown from an aqueous solution consisting of a stoichiometric mixture (ratio 1:1.5:0.5) of H_6_TeO_6_ (Aldrich, 99%) (NH_4_)_2_SO_4_ (Aldrich, 99.99%) and Na_2_SO_4_ (Aldrich, 99%) after evaporation at room temperature.

### Refinement

H atoms of the Te(OH)_6_ group were located in an electron density difference map and were refined with O—H distance restraints of 0.95 (2) Å and a common *U*_iso_ parameter. H atoms of the ammonium groups could not be located and were excluded from the refinement. For the refinement of the occupation factors for N and Na atoms, their sums were restrained to be equal to 1. The highest peak in the final Fourier map is located 0.044 Å from Te2 and the deepest hole 0.43 Å from the same atom.

### Crystal data

**Table:**

Na_0.39_(NH_4_)_1.61_SO_4_·Te(OH)_6_	*F*_000_ = 678.224
*M**_r_* = 357.22	*D*_x_ = 2.418 Mg m^−^^3^
Monoclinic, *P*2_1_/*c*	Mo *K*α radiation λ = 0.71073 Å
Hall symbol: -P 2ybc	Cell parameters from 919 reflections
*a* = 13.690 (1) Å	θ = 2.7–30.1º
*b* = 6.592 (1) Å	µ = 3.30 mm^−^^1^
*c* = 11.345 (1) Å	*T* = 298 K
β = 106.58 (1)º	Parallelepiped, colourless
*V* = 981.26 (19) Å^3^	0.15 × 0.14 × 0.10 mm
*Z* = 4	

### Data collection

**Table:**

Nonius KappaCCD diffractometer	638 reflections with *I* > 3σ(*I*)
Monochromator: graphite	*R*_int_ = 0.000
*T* = 297 K	θ_max_ = 30.2º
φ scans	θ_min_ = 1.6º
Absorption correction: multi-scan(MULABS in PLATON; Spek, 2007)	*h* = −16→14
*T*_min_ = 0.615, *T*_max_ = 0.719	*k* = −7→7
919 measured reflections	*l* = −5→5
849 independent reflections	

### Refinement

**Table:**

Refinement on *F*	Secondary atom site location: difference Fourier map
Least-squares matrix: full	Hydrogen site location: inferred from neighbouring sites
*R*[*F*^2^ > 2σ(*F*^2^)] = 0.032	H-atom parameters constrained
*wR*(*F*^2^) = 0.043	Chebychev polynomial, (Watkin, 1994, *P*rince, 1982) [*w*eight] = 1.0/[A_0_*T_0_(x) + A_1_*T_1_(x) ··· + A_n-1_]*T_n-1_(x)] where A_i_ are the Chebychev coefficients listed belo*w* and x = *F* /*F*max Method = Robust Weighting (*P*rince, 1982); W = [*w*eight] * [1-(delta*F*/6*sigma*F*)^2^]^2^ A_i_ are: 0.527 0.367 0.302
*S* = 0.93	(Δ/σ)_max_ = 0.0001
638 reflections	Δρ_max_ = 0.51 e Å^−^^3^
104 parameters	Δρ_min_ = −1.15 e Å^−^^3^
1 restraint	Extinction correction: None
Primary atom site location: structure-invariant direct methods	

### Fractional atomic coordinates and isotropic or equivalent isotropic displacement parameters (Å^2^)

**Table:**

	*x*	*y*	*z*	*U*_iso_*/*U*_eq_	Occ. (<1)
Te1	0.5000	0.5000	0.0000	0.0099	
Te2	0.0000	1.0000	0.0000	0.0106	
S1	−0.24900 (9)	−0.49139 (17)	−0.2352 (2)	0.0124	
Na1	−0.1448 (2)	0.0149 (2)	−0.3454 (2)	0.0181	0.2590
N1	−0.1448 (2)	0.0149 (2)	−0.3454 (2)	0.0181	0.7410
Na2	−0.3539 (2)	0.0047 (2)	−0.0920 (2)	0.0229	0.1300
N2	−0.3539 (2)	0.0047 (2)	−0.0920 (2)	0.0229	0.8700
O1	0.5309 (3)	0.5871 (6)	−0.1453 (6)	0.0241	
O2	0.4606 (3)	0.2370 (5)	−0.0661 (5)	0.0232	
O3	0.3647 (2)	0.6044 (5)	−0.0656 (5)	0.0174	
O4	−0.1350 (2)	1.0859 (5)	−0.0867 (5)	0.0188	
O5	0.0167 (2)	1.2375 (5)	0.1011 (5)	0.0150	
O6	0.0519 (2)	1.1390 (5)	−0.1165 (6)	0.0174	
O7	−0.1698 (3)	−0.5105 (5)	−0.1149 (6)	0.0221	
O8	−0.3350 (2)	−0.6287 (5)	−0.2355 (5)	0.0160	
O9	−0.2843 (2)	−0.2792 (5)	−0.2508 (5)	0.0202	
O10	−0.2079 (3)	−0.5499 (6)	−0.3357 (6)	0.0213	
H1	0.5496	0.4942	−0.1631	0.0500*	
H2	0.4006	0.2489	−0.1288	0.0500*	
H3	0.3748	0.7041	−0.1257	0.0500*	
H4	−0.1328	1.2273	−0.0942	0.0500*	
H5	−0.0379	1.3302	0.0591	0.0500*	
H6	0.1037	1.0563	−0.1347	0.0500*	

### Atomic displacement parameters (Å^2^)

**Table:**

	*U*^11^	*U*^22^	*U*^33^	*U*^12^	*U*^13^	*U*^23^
Te1	0.00995 (8)	0.00995 (8)	0.00995 (8)	0.00017 (8)	0.00296 (8)	0.00017 (8)
Te2	0.01064 (8)	0.01064 (8)	0.01064 (8)	0.00017 (8)	0.00316 (8)	0.00017 (8)
S1	0.0128 (9)	0.0116 (8)	0.013 (3)	0.0000 (4)	0.0042 (12)	0.0010 (8)
Na1	0.0213 (2)	0.0181 (2)	0.0197 (2)	0.0029 (2)	0.0133 (2)	0.0000 (2)
N1	0.0213 (2)	0.0181 (2)	0.0197 (2)	0.0029 (2)	0.0133 (2)	0.0000 (2)
Na2	0.0233 (2)	0.0199 (2)	0.0285 (2)	−0.0014 (2)	0.0122 (2)	0.0024 (2)
N2	0.0233 (2)	0.0199 (2)	0.0285 (2)	−0.0014 (2)	0.0122 (2)	0.0024 (2)
O1	0.0316 (18)	0.030 (2)	0.016 (2)	0.0127 (15)	0.015 (2)	0.009 (2)
O2	0.0272 (16)	0.0145 (12)	0.020 (4)	0.0006 (12)	−0.006 (2)	−0.0080 (17)
O3	0.0103 (11)	0.0209 (16)	0.021 (4)	0.0017 (11)	0.0045 (16)	0.003 (2)
O4	0.0143 (11)	0.0161 (16)	0.023 (4)	0.0017 (11)	0.0005 (15)	0.0052 (19)
O5	0.0194 (15)	0.0176 (14)	0.008 (3)	0.0032 (12)	0.0042 (19)	−0.0027 (16)
O6	0.0234 (16)	0.0191 (15)	0.014 (3)	0.0045 (13)	0.0119 (19)	0.0034 (17)
O7	0.0190 (18)	0.0192 (18)	0.022 (4)	−0.0001 (12)	−0.004 (2)	0.002 (2)
O8	0.0157 (15)	0.0212 (16)	0.013 (5)	−0.0030 (12)	0.006 (2)	0.002 (2)
O9	0.0167 (16)	0.0146 (13)	0.026 (5)	0.0022 (12)	0.001 (2)	0.001 (2)
O10	0.0189 (18)	0.0247 (16)	0.026 (4)	−0.0032 (14)	0.015 (2)	−0.005 (2)

### Geometric parameters (Å, °)

**Table:**

Te1—H1^i^	2.146	O4—H4	0.937
Te1—O3^i^	1.916 (3)	O5—H5	0.978
Te1—O2^i^	1.905 (4)	O6—H6	0.963
Te1—O1^i^	1.903 (6)	Na1—O6^iii^	2.873 (4)
Te1—O1	1.903 (6)	Na1—O4^iv^	2.937 (6)
Te1—O2	1.905 (4)	Na1—O5^v^	2.947 (4)
Te1—O3	1.916 (3)	Na1—O3^vi^	2.950 (4)
Te1—H1	2.146	Na1—O7^vii^	2.978 (7)
Te2—O5^ii^	1.915 (4)	Na1—O10^viii^	3.008 (4)
Te2—O4^ii^	1.914 (3)	Na1—O9	3.120 (4)
Te2—O6^ii^	1.904 (5)	Na1—O6^iv^	3.267 (6)
Te2—O4	1.914 (3)	Na1—O5^ix^	3.278 (5)
Te2—O5	1.915 (4)	Na2—O9	2.938 (5)
Te2—O6	1.904 (5)	Na2—O8^viii^	2.966 (4)
S1—O7	1.486 (6)	Na2—O4^iv^	3.029 (4)
S1—O8	1.485 (3)	Na2—O10^x^	3.037 (7)
S1—O9	1.474 (3)	Na2—O2^xi^	3.050 (4)
S1—O10	1.460 (6)	Na2—O2^xii^	3.063 (5)
O1—H1	0.715	Na2—O1^xiii^	3.144 (5)
O2—H2	0.924	Na2—O3^ix^	3.164 (5)
O3—H3	0.985	Na2—O1^vi^	3.305 (6)
			
O3^i^—Te1—O2^i^	92.32 (15)	O4^ii^—Te2—O6^ii^	89.85 (18)
O3^i^—Te1—O1^i^	89.1 (2)	O5^ii^—Te2—O4	90.07 (16)
O2^i^—Te1—O1^i^	92.4 (2)	O4^ii^—Te2—O4	179.994
O3^i^—Te1—O1	90.9 (2)	O6^ii^—Te2—O4	90.15 (18)
O2^i^—Te1—O1	87.6 (2)	O5^ii^—Te2—O5	179.994
O1^i^—Te1—O1	179.994	O4^ii^—Te2—O5	90.07 (16)
H1^i^—Te1—O2	103.413	O6^ii^—Te2—O5	88.98 (19)
O3^i^—Te1—O2	87.68 (15)	O4—Te2—O5	89.93 (16)
O2^i^—Te1—O2	179.994	O5^ii^—Te2—O6	88.98 (19)
O1^i^—Te1—O2	87.6 (2)	O4^ii^—Te2—O6	90.15 (18)
O1—Te1—O2	92.4 (2)	O6^ii^—Te2—O6	179.994
H1^i^—Te1—O3	79.615	O4—Te2—O6	89.85 (18)
O3^i^—Te1—O3	179.994	O5—Te2—O6	91.02 (19)
O2^i^—Te1—O3	87.68 (15)	O7—S1—O8	108.7 (3)
O1^i^—Te1—O3	90.9 (2)	O7—S1—O9	108.6 (3)
O1—Te1—O3	89.1 (2)	O8—S1—O9	110.21 (19)
O2—Te1—O3	92.32 (15)	O7—S1—O10	110.6 (3)
O5^ii^—Te2—O4^ii^	89.93 (16)	O8—S1—O10	108.6 (3)
O5^ii^—Te2—O6^ii^	91.02 (19)	O9—S1—O10	110.1 (3)

Symmetry codes: (i) −*x*+1, −*y*+1, −*z*; (ii) −*x*, −*y*+2, −*z*; (iii) −*x*, *y*−3/2, −*z*−1/2; (iv) *x*, *y*−1, *z*; (v) *x*, −*y*+3/2, *z*−1/2; (vi) −*x*, *y*−1/2, −*z*−1/2; (vii) *x*, −*y*−1/2, *z*−1/2; (viii) *x*, *y*+1, *z*; (ix) −*x*, −*y*+1, −*z*; (x) *x*, −*y*−1/2, *z*+1/2; (xi) *x*−1, *y*, *z*; (xii) −*x*, −*y*, −*z*; (xiii) *x*−1, *y*−1, *z*.

### Hydrogen-bond geometry (Å, °)

**Table:**

*D*—H···*A*	*D*—H	H···*A*	*D*···*A*	*D*—H···*A*
O2—H2···O9^xiv^	0.924 (4)	1.787 (4)	2.700 (6)	169.2 (2)
O3—H3···O8^xv^	0.985 (5)	1.871 (5)	2.799 (7)	155.7 (2)
O4—H4···O7^xvi^	0.937 (3)	1.798 (3)	2.706 (7)	162.4 (2)
O6—H6···O10^xv^	0.963 (4)	1.706 (4)	2.658 (6)	169.5 (3)
N1—···.O6^iii^	.	.	2.873 (4)	.
N1—···.O4^iv^	.	.	2.937 (6)	.
N1—···.O5^v^	.	.	2.947 (4)	.
N1—···.O3^vi^	.	.	2.950 (4)	.
N1—···.O7^vii^	.	.	2.978 (7)	.
N1—···.O10^viii^	.	.	3.008 (4)	.
N2—···.O9	.	.	2.938 (5)	.
N2—···.O8^viii^	.	.	2.966 (4)	.
N2—···.O4^iv^	.	.	3.029 (4)	.
N2—···.O10^x^	.	.	3.037 (7)	.
N2—···.O2^xi^	.	.	3.050 (4)	.
N2—···.O2^xii^	.	.	3.063 (5)	.

Symmetry codes: (xiv) −*x*, *y*+1/2, −*z*−1/2; (xv) −*x*, *y*+3/2, −*z*−1/2; (xvi) *x*, *y*+2, *z*; (iii) −*x*, *y*−3/2, −*z*−1/2; (iv) *x*, *y*−1, *z*; (v) *x*, −*y*+3/2, *z*−1/2; (vi) −*x*, *y*−1/2, −*z*−1/2; (vii) *x*, −*y*−1/2, *z*−1/2; (viii) *x*, *y*+1, *z*; (x) *x*, −*y*−1/2, *z*+1/2; (xi) *x*−1, *y*, *z*; (xii) −*x*, −*y*, −*z*.
